# Accuracy of international growth charts to assess nutritional status in children and adolescents: a systematic review

**DOI:** 10.1590/1984-0462/2022/40/2021016

**Published:** 2022-04-04

**Authors:** Mariane Helen de Oliveira, Débora dos Santos Pereira, Daiane Sousa Melo, Jessica Cumpian Silva, Wolney Lisboa Conde

**Affiliations:** a Universidade de São Paulo, São Paulo, SP, Brazil.

**Keywords:** Child, Adolescent, Nutrition assessment, Growth charts, Stature by age, Body mass index, Criança, Adolescente, Avaliação nutricional, Gráficos de crescimento, Estatura-idade, Índice de massa corporal

## Abstract

**Objective:**

To verify, through a systematic review, the accuracy of nutritional assessment in children and adolescents using the length/height-for-age and BMI-for-age growth charts of the Centers for Disease Control and Prevention (CDC) (2000), the World Health Organization (WHO) (2006/2007) and the International Obesity Task Force (IOTF) (2012).

**Data source:**

We selected articles from the databases *Medical Literature Analysis and Retrieval System Online* (MEDLINE), through PubMed, National Library of Medicine and The National Institutes of Health (NIH), Scientific Electronic Library Online (SciELO) and Virtual Health Library (VHL). The following descriptors were used for the search: “Child”, “Adolescent”, “Nutritional Assessment”, “Growth Chart”, “Ethnic Groups”, “Stature by age”, “Body Mass Index”, “Comparison”, “CDC”, “WHO”, and “IOTF”. The selected articles were assessed for quality through the Quality Assessment Tool for Observational Cohort and Cross-Sectional Studies of the NIH.

**Data synthesis:**

Thirty-three studies published between 2007 and 2020 were selected and, of these, 20 presented good quality, 12 presented fair quality and one presented poor quality. For children under five years old, the WHO length/height-for-age growth charts were shown appropriate for children from Argentina, South Africa, Brazil, Gabon, Qatar, Pakistan and the United States. For those five years old and older, the WHO BMI-for-age growth charts were accurate for the Brazilian and Canadian populations, while the IOTF growth charts were accurate for the European populations.

**Conclusions:**

There are difficulties in obtaining international growth charts for children from 5 years old and older that go along with a long period of growth, and which include genetic, cultural and socioeconomic differences of multiethnic populations who have already overcome the secular trend in height.

## Resumo

**Objetivo:**

Verificar, por meio de uma revisão sistemática, a acurácia da avaliação nutricional em crianças e adolescentes com base nas curvas de crescimento de comprimento/altura para a idade e índice de massa corporal para a idade do *Centers for Disease Control and Prevention* (CDC) (2000), Organização Mundial da Saúde (OMS) (2006/2007) e *International Obesity Task Force* (IOTF) (2012).

**Fontes de dados:**

Os artigos foram selecionados nas bases de dados *Medical Literature Analysis and Retrieval System Online* (MEDLINE), via PubMed, *National Library of Medicine* e *The National Institutes of Health* (NIH), *Scientific Electronic Library Online* (SciELO) e Biblioteca Virtual em Saúde (BVS). Os seguintes descritores foram utilizados na busca: “criança’’, “adolescente’’, “avaliação nutricional”, “gráficos de crescimento”, “grupos étnicos”, “estatura-idade”, “índice de massa corporal”, “comparação”, “CDC”, “OMS” e “IOTF”. Os artigos selecionados tiveram sua qualidade avaliada por meio da escala *Quality Assessment Tool for Observational Cohort and Cross-Sectional Studies* da NIH.

**Síntese dos dados:**

Trinta e três estudos publicados entre 2007 e 2020 foram selecionados e, deles, 20 tinham boa qualidade, 12 tinham qualidade razoável e 1 tinha qualidade insatisfatória. Para menores de cinco anos, as curvas de comprimento/altura para a idade da OMS demonstraram ser apropriadas para as populações de Argentina, África do Sul, Brasil, Gabão, Catar, Paquistão e Estados Unidos. Para maiores de cinco anos, as curvas de IMC para a idade da OMS apresentaram-se acuradas para as populações brasileira e canadense, enquanto as curvas do IOTF se apresentaram acuradas para as populações europeias.

**Conclusões:**

Para maiores de cinco anos, há dificuldade de se obterem curvas internacionais que possam atender a um período tão longo de crescimento e que incluam diferenças genéticas, culturais e socioeconômicas de populações multiétnicas que já tenham superado a tendência secular de crescimento.

## INTRODUCTION

For decades, the precision in assessing the growth of children and adolescents has been the object of study by several researchers, who use anthropometry and growth charts to monitor the evolution of growth changes and to assess the nutritional status of children under 20 years of age.^1^ These growth charts were created based on longitudinal and/or cross-sectional studies with samples of children and adolescents considered a reference or standard.^2,3^ They express distributions in percentiles or Z scores and are considered quite sensitive for the assessment of nutritional status, enabling interventions and the prevention of health problems.^2,3^

Different growth charts have been proposed by some institutions and organizations over the years for use in the world population, through studies with national or international samples and with different inclusion criteria.^4^ Among these, the growth charts by the Centers for Disease Control and Prevention (CDC) (2000), the World Health Organization (WHO) (2006/2007) and the International Obesity Task Force (IOTF) (2012) stand out.^5–8^

The CDC growth charts were drawn up in the 2000s based on five national surveys conducted in the United States.^5,9^ They are expressed in percentiles and are specific by sex and age group.^5,9^ For children under three years of age, there are growth charts of length-for-age, weight-for-age and head circumference-for-age.^5,9^ For children under five, there is a weight-for-height growth chart, and for children and adolescents aged between two and 20 years, there are growth charts representing stature-for-age, weight-for-age and body mass index (BMI) for age.^5,9^

The WHO growth charts for children under the age of five were developed in 2006 based on the Multicenter Growth Reference Study, whose goal was to describe the growth of healthy children.^6^ This work was conducted in six countries: Brazil (Pelotas), United States (Davis), Ghana (Accra), Norway (Oslo), India (New Delhi) and Oman (Muscat) with children considered standard, that is, who lived in socio-environmental and economic conditions ideal for an adequate development.^2,6^ These growth charts were constructed based on longitudinal (from birth to two years old) and cross-sectional samples with children aged 18 to 71 months.^6^ For children aged five years or more and adolescents aged up to 20 years, the construction of the growth charts was based on the cross-sectional study of the National Center for Health Statistics (NCHS/1977), whose only study population was from the United States.^3,7,10^ For the construction of these growth charts, the WHO specialists committee remodeled the 1997 NCHS data, keeping only non-obese children and adolescents who had reached expected heights for their age and adding growth patterns data for under-fives aged 18 to 71 months.^3,7,10^ The addition of these data smoothed the growth charts, creating a smooth transition at five years of age and at the end of adolescence, with adjustment to the overweight and obesity cutoff points recommended for adults.^3,7,10^

The WHO growth charts are expressed in percentiles or Z-scores and are specific for sex and age group.^6,7^ For children under five, there are head circumference-for-age and weight-for-height growth charts.2,6 For children under 10 years old there is the weight-for-age growth chart and, for children and adolescents under 20 years there are the length/height-for-age and BMI-for-age growth charts.2,3,6,7,10

Still in the 2000s, the IOTF developed the BMI-for-age growth charts for children and adolescents aged between two and 20 years, with BMI values of 25 and 30 kg/m^2^ for 18 years, suggesting classifications distributed by age and sex, as well as overweight and obesity classifications.^11^ In 2012, after studies showed divergences in the WHO growth reference (2006/2007) in some populations, the IOTF released an update of its cutoff points using international samples and proposed these for the BMI, which resulted in six different classifications similar to WHO’s, ranging from severe thinness to morbid obesity.^8^

The two main anthropometric indicators used in the assessment of children and adolescents are length/height-for-age and BMI-for-age.^2,3,10^ These indicators have the following objectives, respectively: a) to show the linear trajectory of growth, being fundamental in the detection of stunting; b) to detect underweight or overweight.^2,3,10^ The cutoff points of the CDC (2000), the WHO (2006/2007) and the IOTF (2012), in percentiles, for the length/height -for-age and BMI-for-age indicators are shown in Chart 1.

**Chart 1:** Cut-off points, in percentiles, of indicators of length/height-for-age and BMI-for-age from the Centers for Disease Control and Prevention (2000), the World Health Organization (2006/2007) and the International Obesity Task Force (2012).

Indicator	Age group	Nutritional status	WHO*(♂/♀)	CDC** (♂/♀)	IOTF**	
					♂	♀
Length/height-for-age	0<20 years	Stunting	p<3.00	p<5.00	--	--
		Normal height	p≥3.00	p≥5.00	--	--
BMI-for-age	0<5 years	Severe thinness	p<0.10	--	--	--
		Thinness	p≥0.10; p<3.00	p<5.00	p<15.50	p<6.50
		Normal weight	p≥3.00; p≤85.00	p≥5.00; p<85.00	p≥15.50; p<90.50	p≥16.50; p<89.30
		Risk of overweight	p>85.00; p≤97.00	--	--	--
		Overweight	p>97.00; p≤99.90	p≥85.00; p<95.00	p≥90.50; p<98.90	p≥89.30; p<98.60
		Obesity	p>99.90	p≥95.00	p≥98.90; p<99.83	p≥98.60; p<99.76
		Morbid obesity	--	--	p≥99.83	p≥99.76
	5<20 years	Severe thinness	p<0.10	--	--	--
		Thinness	p≥0.10; p<3.00	p<5.00	p<15.50	p<16.50
		Normal weight	p≥3.00; p≤85.00	p≥5.00; p<85.00	p≥15.50; p<90.50	p≥16.50; p<89.30
		Overweight	p>85.00; p≤97.00	p≥85.00; p<95.00	p≥90.50; p<98.90	p≥89.30; p<98.60
		Obesity	p>97.00; p≤99.90	p≥95.00	p≥98.90; p<99.83	p≥98.60; p<99.76
		Morbid obesity	p>99.90	--	p≥99.83	p≥99.76

CDC: Centers for Disease Control and Prevention; WHO: World Health Organization; IOTF: International Obesity Task Force Source: adapted from CDC (2000), WHO (2006/2007) and IOTF (2012).^5–8^ Length: measured with the child lying down (<2 years of age). Height: measured with the child/adolescent standing (≥2 years old). ♂: male children and adolescents ♀: female children and adolescents. p: percentile. *Body mass index-for-age reference values from birth. **Body mass index-for-age reference values from two-year-old. -- not applicable (no cutoff points or references for these classifications).

WHO recommends its own growth charts (2006/2007) for international use, and they have been adopted in health and nutrition programs in more than 140 countries, including Brazil.^4^ However, some studies have shown divergent comparisons between the national growth charts and the WHO growth charts.4 Examples are places like the United Kingdom, Poland, Norway, Germany, Hong Kong, Iran, United Arab Emirates and South Africa.^4,12,13,14,15^ For this reason, the United Kingdom created growth charts for certain ages based on the joining of the WHO growth references with local data, while countries such as China, Bolivia, Denmark, Norway and Belgium, have not used the WHO growth charts widely due to divergences in growth parameters of their populations when compared to the reference growth charts.4,15

The methodological differences in establishing cutoff points between the CDC, WHO and IOTF references involve population composition and modeling of descriptive parameters of the anthropometric index and cutoff points.^2,4,9,16^ These differences generate effects on the accuracy of nutritional classification and, by extension, make diagnosis and comparison of prevalence difficult.^2,16^

Some authors justify that these growth charts should be based on local populations, since there are genetic, cultural and socioeconomic differences that impact the processes of physical growth and biological maturation, which result in different growth profiles and BMI.^12,15^ Furthermore, variation in body composition between children and adolescents of different ethnicities has been an obstacle to the determination of an international standard for classification of nutritional status.^16^ Thus, the objective of this study was to verify, through a systematic review, the accuracy of nutritional assessment in children and adolescents based on the growth charts recommended for international use of length/height-for-age and BMI-for-age by the CDC (2000), WHO (2006/2007) and IOTF (2012).

## METHOD

This study is characterized as a systematic literature review, designed in accordance with the recommendations proposed by the Preferred Reporting Items for Systematic Reviews and Meta-Analyses (PRISMA).^17^ This project was registered in the International Prospective Register of Systematic Reviews (PROSPERO) under protocol CRD42020215498, and the data and outlines of this review can be accessed at www.crd.york.ac.uk/PROSPERO/display_record.asp?ID=CRD42020215498.

The Participants, Intervention, Comparison, Outcome, Study Design (PICOS) strategy was applied for the selection of studies. We considered the studies that evaluate: P (children and adolescents), I (length/height-for-age and/or BMI-for-age growth charts recommended for international use), C (national and/or international growth charts), O (nutritional status), S (cohort, cross-sectional).

Two independent researchers consulted articles published in Portuguese, Spanish and English between 2000 and 2020 in the Electronic Medical Literature Analysis and Retrieval System Online (MEDLINE) databases, via PubMed, National Library of Medicine and The National Institutes of Health, Scientific Electronic Library Online (SciELO) and Virtual Health Library (VHL). In the search strategy, the terms of the Medical Subject Headings (MeSH) and the Health Sciences Descriptors (DeCS) used were: “child’’, “adolescent”, “nutritional assessment”, “growth charts”, “ethnic groups”, “stature by age”, “body mass index”, “comparison”, “CDC”, “WHO” and “IOTF” (in combined form, in both Portuguese and in English languages).

Studies were considered eligible for inclusion when they met the following criteria: a) evaluated the CDC (2000) and/or WHO (2006/2007) length/height-for-age growth charts in children and/or adolescents; and/or b) evaluated the BMI-for-age growth charts of the CDC (2000) and/or the WHO (2006/2007) and/or the IOTF (2012) in children and/or adolescents. The selection of evidence was restricted to original articles, excluding review studies, experimental studies with animals, case reports, duplicate studies and studies published in languages other than those mentioned above.

The selection was first conducted by means of titles, then abstracts and, finally, full reading. The three steps were performed by two evaluators, who decided on inclusion in each step based on the eligibility criteria. Each evaluator independently decided for “inclusion” or “exclusion” and any divergent results were analyzed by a third evaluator. Eligible studies had their data extracted independently by two authors, who organized them in instruments built for this purpose, following methodological recommendations and contemplating the following items: identification of original article, study design, study population, sample size and main results related to the evaluated indicators/references.

The quality of the articles was assessed by adapting the Quality Assessment Tool for Observational Cohort and Cross-Sectional Studies, appropriate for observational studies, by the NIH.^18^ This instrument suggests the classification of quality as good, fair, and poor based on the analysis of 14 items.^18^ To assess the studies included in this review, eight items of this scale were used, referring to study objectives, study population, selection criteria, statistical power of the sample, intervention/exposure measures, loss to follow-up and outcome.

## RESULTS AND DISCUSSION

**Figure 1:**
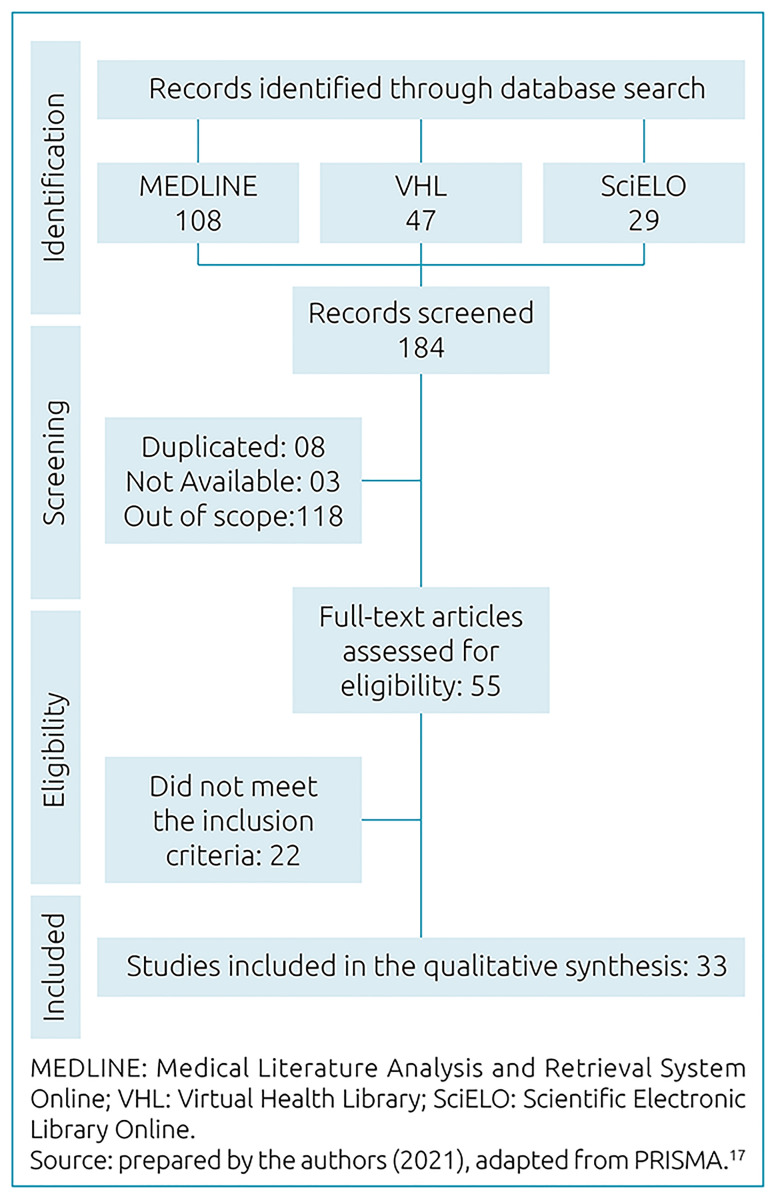
Study selection flowchart. São Paulo, SP, Brazil, 2021.

In October 2020, 184 articles published between 2000 and 2020 were identified in the databases. After selection by title, 91 studies were excluded, with 93 articles remaining for the abstract analysis. Fifty-five articles were selected for full reading, of which 33, published between 2007 and 2020, were included in the synthesis of evidence for this review.^12,16,19–49^ Figure 1 shows the process of article selection in its different stages and respective numbers of studies retrieved.

Studies from several countries were identified addressing the application of international growth charts in their populations. To present the results of this review, we grouped the synthesis of findings of the 33 evidences of studies that evaluated growth charts for children under five years old (Chart 2), for children from five years old (Chart 3) and for children and adolescents aged zero to 20 years old (Chart 4).

**Chart 2:** Identification and characteristics of studies that evaluated the accuracy of growth charts for children under five years of age.

Authors and year of publication	Study design and population	Main results						
		Length/height-for-age			BMI-for-age			
		NAT	CDC	WHO	NAT	CDC	WHO	IOTF
Alfaro et al., 2008^19^	Cross-sectional — Argentina (Jujuy): 4,678 girls and 4,414 boys aged between 0 and 5 years	Ө	X	✓	--	--	--	--
Silveira et al., 2011^20^	Cross-sectional — Brazil (Porto Alegre): 203 hospitalized boys and 134 hospitalized girls aged between 0 and 5 years	--	X	✓	--	--	--	--
Bagni et al., 2012^21^	Cross-sectional — Brazil: (Rio de Janeiro): Height-for-age: 299 girls and 339 boys; BMI-for-age: 254 girls and 286 boys aged between 1 and 5 years	--	X	✓	--	X	✓	--
Pereira et al., 2010^22^	Cross-sectional — Brazil: (Rio de Janeiro): 157 girls and 155 boys aged 2-3 years	--	X	✓	--	--	--	--
Mei et al., 2008^23^	Cross-sectional — United States: 3,920 children aged 0-5 years	--	Ө	✓	--	X	✓	--
Bosman et al., 2011^24^	Cross-sectional — South Africa: 714 girls and 798 boys aged 1-5 years	--	X	✓	--	X	✓	--
Schwarz et al., 2008^25^	Longitudinal — Gabon (Lambaréné): 150 girls and 139 boys evaluated at birth, at 3, 9 and 15 months of age	--	X	✓	--	--	--	--
Soliman et al., 2011^26^	Longitudinal — Qatar: 150 girls and 150 boys evaluated at birth, at 2, 4, 6, 12 and 18 months.	Ө	X	✓	--	--	--	--
Perera et al., 2014^27^	Longitudinal — Sri Lanka: 241 girls and 244 boys evaluated at 2, 4, 6, 9, and 12 months.	✓	X	X	--	--	--	--
Nuruddin et al., 2009^28^	Cross-sectional — Pakistan: 2,584 Children aged 0 to 35 months National Survey: 721 girls and 812 boys Thatta survey: 494 girls and 557 boys	Ө	--	✓	--	--	--	--
Onis et al., 2007^29^	Longitudinal — Canada, United States and European countries: children aged 0-12 months	--	Ө*	✓*	--	Ө*	✓*	--

Source: prepared by the authors (2021). BMI: body mass index; NAT: national growth charts for the country of study; CDC: Centers for Disease Control and Prevention; WHO: World Health Organization; IOTF: International Obesity Task Force; ✓: appropriate for the study population; Ө: reasonable for the study population; X: inadequate for the study population; --: not applicable, evaluation of indicator/reference not carried out in the study; *growth chart accuracy only applicable to the US population.

**Chart 3:** Identification and characteristics of studies that assessed the accuracy of growth charts for children and adolescents from five years old.

Authors and year of publication	Study design and population	Main results						
		Length/height-for-age			BMI-for-age			
		NAT	CDC	WHO	NAT	CDC	WHO	IOTF
Mohammadi et al., 2020^30^	Cross-sectional — Iran: 11,797 girls and 10,921 boys aged between 6 and 18 years	--	--	--	✓	X	--	--
Esmaili et al., 2019^31^	Cross-sectional — Northern Iran (Babol): 2,090 girls and 1,993 boys aged between 7 and 11 years	--	--	--	✓	X	X	--
Ma et al., 2010^32^	Cross-sectional — China: 115,374 girls and 116,766 boys aged between 7 and 18 years	--	--	--	✓	X	X	--
Cavazzotto et al., 2014^16^	Cross-sectional — Brazil (Maringá, Rio Claro, Guarapuava and Londrina): 778 girls and 863 boys aged between 6 and 13 years	--	--	--	--	--	Ө	Ө
Silva et al., 2018^33^	Cross-sectional — Brazil (São José/SC): 613 girls and 519 girls aged between 14 and 19 years	--	--	--	✓	--	✓	X
Roman et al., 2015^34^	Cross-sectional — Brazil (Cascavel): 2,729 girls aged between 9 and 17 years	--	--	--	✓	X	✓	--
Romagna et al., 2010^35^	Cross-sectional — Brazil (Canoas, Porto Alegre): 155 girls and 117 boys aged between 5 and 18 years	--	--		--	X	✓	--
Silva et al., 2010^36^	Cross-sectional — Brazil (North, Northeast, Midwest, Southeast and South): 18,326 girls and 23,328 boys aged between 7 and 17 years	Ө	Ө	Ө	Ө	X	✓	--
Cossio-Bolaños et al., 2015^37^	Cross-sectional — Peru (Arequipa): 138 girls and 181 boys aged between 12 and 18 years	✓	X	--	✓	X	--	--
Valerio et al., 2017^38^	Cross-sectional — Italy: 3,061 girls and 3,009 boys aged between 5 and 17 years	--	--	--	✓	--	Ө	✓
Minghelli et al., 2014^39^	Cross-sectional — Portugal (Algarve): 529 girls and 437 boys aged between 10 and 16 years	--	--	--	--	✓	X	✓
Wózniacka et al., 2018^40^	Cross-sectional — Poland (Kraków): 1,674 girls and 1,731 boys aged between 5 and 14 years	--	--	--	✓	X	--	✓
Regecová et al., 2018^41^	Cross-sectional — Slovakia: 19,220 girls and 19,472 boys aged between 7 and 18 years	✓	--	X	✓	--	X	Ө
Kakinami et al., 2012^42^	Cross-sectional — Canada (Quebec): 1,262 girls and 1,204 boys aged 9, 13 and 16 years old	--	--	--	--	✓	✓	--
Moselakgomo e van Staden, 2017^43^	Cross-sectional — South Africa (Mpumalanga and Limpopo): 683 girls and 678 boys aged between 9 and 13 years	--	--	--	--	Ө	--	Ө

Source: prepared by the authors (2021). BMI: body mass index; NAT: national growth charts for the country of study; CDC: Centers for Disease Control and Prevention; WHO: World Health Organization; IOTF: International Obesity Task Force; ✓: appropriate for the study population; Ө: reasonable for the study population; X: inadequate for the study population; --: not applicable, evaluation of indicator/reference not carried out in the study.

**Chart 4:** Identification and characteristics of studies that assessed the accuracy of growth charts for children and adolescents aged between 0 and 20 years.

Authors and year of publication	Study design and population	Main results						
		Length/ height-for-age			BMI-for-age			
		NAT	CDC	WHO	NAT	CDC	WHO	IOTF
Rosario et al., 2011^12^	Cross-sectional — Germany: 8,408 girls and 8,671 boys aged between 0 and 18 years	✓	X	X	--	--	--	--
Hughes et al., 2014^44^	Longitudinal and cross-sectional — Australia: 2,979 girls and 3,117 boys aged between 2 and 16 years	✓	X	X	--	--	--	--
Oliveira et al., 2013^45^	Longitudinal — Brazil (Porto Alegre): 54 girls and 64 boys aged between 2 and 19 years+B29	--	--	--	--	X	✓	--
Zong e Li, 2013^46^	Cross-sectional — China: 47,213 girls and 47,089 boys aged between 0 and 18 years	✓	--	X	✓	--	X	--
Al Herbish et al., 2009^47^	Cross-sectional — Saudi Arabia (SA) (13 regions): 17,399 girls and 17,880 boys aged between 0 and 19 years	--	--	--	✓	X	X	--
El Mouzan et al., 2008^48^	Cross-sectional — Saudi Arabia (SA) (13 regions): 17,399 girls and 17,880 boys aged between 0 and 19 years	✓	X	--	✓	X	--	--
Wilde et al., 2015^49^	Longitudinal — Netherlands (South Asian immigrants): 2,198 girls and 2,195 boys aged 0-20 years	✓	--	X	--	--	--	--

Source: prepared by the authors (2021). BMI: body mass index; NAT: national growth charts for the country of study; CDC: Centers for Disease Control and Prevention; WHO: World Health Organization; IOTF: International Obesity Task Force; ✓: appropriate for the study population; Ө: reasonable for the study population; X: inadequate for the study population; --: not applicable, evaluation of indicator/reference not carried out in the study.

For children under five years old, studies show that the WHO length/height-for-age charts performed better in detecting stunting when compared to CDC growth charts.19-26,28,29 For this reason, the authors recommended the WHO growth charts for populations in Argentina, South Africa, Brazil, Gabon, Qatar, Pakistan and the United States.^19–26,28,29^ However, for the population of Sri Lanka, researchers state the need for further studies, since children in that country presented lower height when compared to the WHO’s standards population.^27^

Regarding the BMI-for-age charts in children under five years old, the WHO diagnosed more children with underweight than the CDC for the US population and more overweight and obese children in South Africa and Brazil, which indicates that they are more appropriate for these populations.^21,24,29^ Some authors argue that the WHO has constructed growth charts for children under five years old based on multiethnic children who had adequate health and nutrition conditions and who received exclusive breastfeeding with until at least three or four months of age, and complementary feeding based on legumes, meat, eggs, fruits and vegetables, with partial breastfeeding, until the 12th month of life or more, which allows these growth charts to be applied internationally and to early diagnose stunting, overweight and obesity, being more accurate than those of the CDC.^21,24,26^

For children and adolescents aged five years or more, studies show that the WHO height-for-age charts have similar values only for the Brazilian population.^36^ Immigrants from South Asia living in the Netherlands had lower height-for-age values than WHO’s standard population, while the populations of Australia, Slovakia and Germany presented higher height values, which indicates that this international reference would not adequately detect stunting for children and adolescents (≥5 years) of these populations.^12,41,44,49^ Similar results were found by Bonthuis et al. in a study that evaluated 18 national height-for-age charts from 28 European countries and compared them with those of the CDC, WHO, and Euro-Growth.^50^ The authors report that these national European growth charts showed a positive secular trend in height, which has been observed since 1850, and that this secular trend has slowed down or even reached a plateau since the 1980s/1990s in many northern European countries, as well as in Italy and the United States.^50^ In addition, the authors reinforce that, although these divergences are associated with genetic and geographic factors, they are strongly affected by the secular trend in height, and that height growth charts constructed with data collected before 1990, including those from the CDC and WHO/2007, produced mean heights generally lower than those in growth charts developed more recently.^50^ Therefore, they advocate the use of specific growth charts for the European population based on recent national data.^50^

Regarding the WHO/2007 BMI-for-age charts for the Brazilian population, they were adequate for diagnosing overweight and obesity, being similar to the Brazilian national growth charts (Conde & Monteiro), and showing substantial agreement with those of the IOTF.^16,33,34,45^ From another perspective, for the Asian populations of China, Saudi Arabia and Iran, there is great variation between the WHO and national growth charts. When compared to the WHO standard/reference populations, Chinese boys present higher weight values and Chinese girls lower weight values, with significant variations in some age groups, while children and adolescents from Saudi Arabia present higher percentile values.^30–32,46,47^

Regarding the IOTF growth charts, for the European populations of Slovakia, Italy, Poland and Portugal, they showed the best performance for screening overweight and obesity, while for the population of South Africa they had the best screening for underweight.^38–41^.^43^ Regarding the CDC BMI-for-age growth charts, their values were similar to those of WHO/2007 for the Canadian population and similar to those of the IOTF for the Portuguese population. On the other hand, they diagnosed more overweight in South Africa and overestimated the diagnoses of overweight, obesity and underweight in Saudi Arabia and underweight in Brazil, while underestimating the diagnoses of overweight in Brazil and obesity in Iran.^30,34,39^ .^43^.^48^

These variations in nutritional diagnosis caused by different BMI-for-age growth charts are in line with the findings of a study conducted by Li et al. with the population of the United States.^51^ In their study. although there was a substantial agreement between the CDC, IOTF and WHO growth charts for the classification of nutritional status of adolescents, those of the IOTF classified more overweight compared to other international references, while the WHO classified more adolescents as overweight and less as obese compared to CDC.^51^ From another perspective, in a study conducted in El Salvador by Pérez et al. with children aged six to nine years, despite the strong agreement between the WHO and IOTF growth charts, the WHO growth reference classifies more overweight and obese children than the IOTF.^52^

Overall, the CDC BMI-for-age growth charts underperformed for screenings of nutritional diagnoses than the growth charts by WHO and IOTF. However, there is still controversy as to which growth reference would be more appropriate for international use, especially for children from five years old and over. Some authors argue that the WHO/2007 growth reference consists of a non-obese sample of children in the United States aged 1-24 years with data collected from 1963 to 1974, being a reference population that represents a healthier group and, therefore, more sensitive to diagnoses of overweight.^7,52^ However, other authors argue that the use of a single population in the modeling of growth charts makes them not suitable for international use and, therefore, they suggest the application of IOTF growth charts, as they were developed by combining the most recent BMI data of children aged 2-18 years from six nationally representative surveys from 1963 to 1993.^11,52^

**Figure 2.**
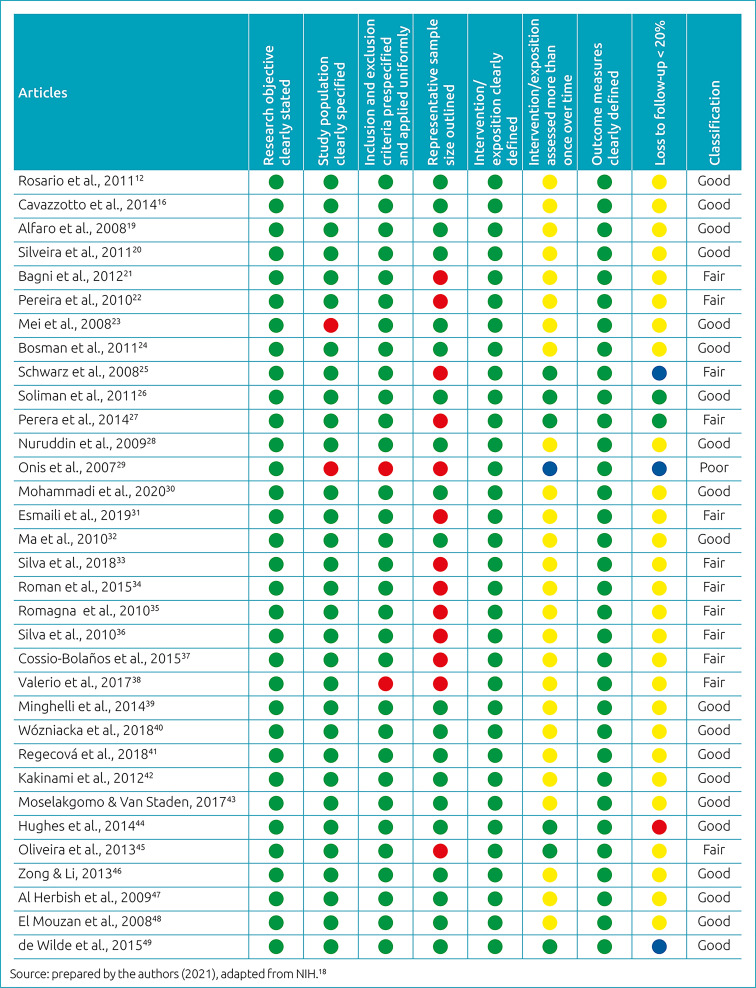
Quality analysis of the articles included in this review.

Regarding the quality of the selected studies, it was considered excellent, with most studies classified as having good methodological quality, as shown in Figure 2. For this assessment, the NIH scale specific for observational studies was used, which is suitable for this type of design, as it assesses objectives of the study, methodological aspects and coherence of results.^18^ Some studies included in this review had limitations such as the absence of sample size and statistical power in cross-sectional studies and the loss to follow-up in cohort studies, although such restrictions have not influenced in the results of this review, given the good methodological quality achieved.

This systematic review allowed, for the first time, the identification and assessment of accuracy of the length/height-for-age international growth charts by CDC and WHO, and BMI-for-age growth charts by CDC, WHO and IOTF in 20 countries from five different continents. Per this investigation, for children under five years old, the WHO length/height-for-age growth charts were proven more accurate than those of the CDC and, therefore, more appropriate for use in the populations of Argentina, South Africa, Brazil, Gabon, Qatar, Pakistan and the United States; the WHO BMI-for-age growth charts also showed better screenings of nutritional status when compared to the CDC, being recommended for the populations of the United States, South Africa and Brazil.

On the other hand, for children from five years old, there is great variation in agreements. The WHO height-for-age charts showed similar patterns for the Brazilian population, while South Asian immigrants living in the Netherlands had lower height values than WHO’s standard population, and the populations of Australia, Slovakia and Germany had higher height values, which indicates that this international reference does not detect stunting adequately. Regarding BMI-for-age, WHO growth charts were accurate for the Brazilian and Canadian populations, while IOTF growth charts were more accurate for the populations of Slovakia, Portugal, Italy and Poland, and CDC growth charts were accurate only for Portugal and Canada. Regarding China, Iran and Saudi Arabia, the authors suggest the use of national growth charts and, for South Africa, they point out the need for further studies to determine the most accurate international growth reference.

The explanation for the international recommendation of the WHO’s reference only for children under five years of age is its modeling and construction, which involved multiethnic populations in environmental and health conditions adequate for their development. Therefore, when it is applied, it presents satisfactory agreements for nutritional status assessment. The opposite is observed when the WHO growth reference is applied to children and adolescents from five years old. This is because the modeling and population used were different, resulting in divergences in nutritional status assessment in several countries, hence its use not widely indicated.

In summary, the international growth charts for children and adolescents from five years old have limitations, since the differences between models and the composition of samples in the construction of growth charts did not allow an international standard for classification of nutritional status. It is difficult to obtain growth charts for international use that can go along with a long period of growth and which include genetic, cultural, socioeconomic and body composition differences of multiethnic children and adolescents who have already overcome the secular trend in height.
